# Histone demethylase JARID1C/KDM5C regulates Th17 cells by increasing IL-6 expression in diabetic plasmacytoid dendritic cells

**DOI:** 10.1172/jci.insight.172959

**Published:** 2024-06-24

**Authors:** Christopher O. Audu, Sonya J. Wolf, Amrita D. Joshi, Jadie Y. Moon, William J. Melvin, Sriganesh B. Sharma, Frank M. Davis, Andrea T. Obi, Rachel Wasikowski, Lam C. Tsoi, Emily C. Barrett, Kevin D. Mangum, Tyler M. Bauer, Steven L. Kunkel, Beth B. Moore, Katherine A. Gallagher

**Affiliations:** 1 Section of Vascular Surgery, Department of Surgery, and; 2 Department of Microbiology and Immunology, University of Michigan Medical School, Ann Arbor, Michigan, USA.; 3 Department of Medicinal Chemistry, College of Pharmacy, University of Michigan, Ann Arbor, Michigan, USA.; 4 Department of Pathology, School of Medicine, University of Michigan Medical School, Ann Arbor, Michigan, USA.

**Keywords:** Immunology, Inflammation, Adaptive immunity, Dendritic cells, Epigenetics

## Abstract

Plasmacytoid dendritic cells (pDCs) are first responders to tissue injury, where they prime naive T cells. The role of pDCs in physiologic wound repair has been examined, but little is known about pDCs in diabetic wound tissue and their interactions with naive CD4^+^ T cells. Diabetic wounds are characterized by increased levels of inflammatory IL-17A cytokine, partly due to increased Th17 CD4^+^ cells. This increased IL-17A cytokine, in excess, impairs tissue repair. Here, using human tissue and murine wound healing models, we found that diabetic wound pDCs produced excess IL-6 and TGF-β and that these cytokines skewed naive CD4^+^ T cells toward a Th17 inflammatory phenotype following cutaneous injury. Further, we identified that increased IL-6 cytokine production by diabetic wound pDCs is regulated by a histone demethylase, Jumonji AT-rich interactive domain 1C histone demethylase (JARID1C). Decreased JARID1C increased IL-6 transcription in diabetic pDCs, and this process was regulated upstream by an IFN-I/TYK2/JAK1,3 signaling pathway. When inhibited in nondiabetic wound pDCs, JARID1C skewed naive CD4^+^ T cells toward a Th17 phenotype and increased IL-17A production. Together, this suggests that diabetic wound pDCs are epigenetically altered to increase IL-6 expression that then affects T cell phenotype. These findings identify a therapeutically manipulable pathway in diabetic wounds.

## Abstract 2

Histone demethylase JARID1C regulates IL-6 in diabetic wound plasmacytoid dendritic cells, leading to increased inflammatory Th17 cells.

## Graphical abstract

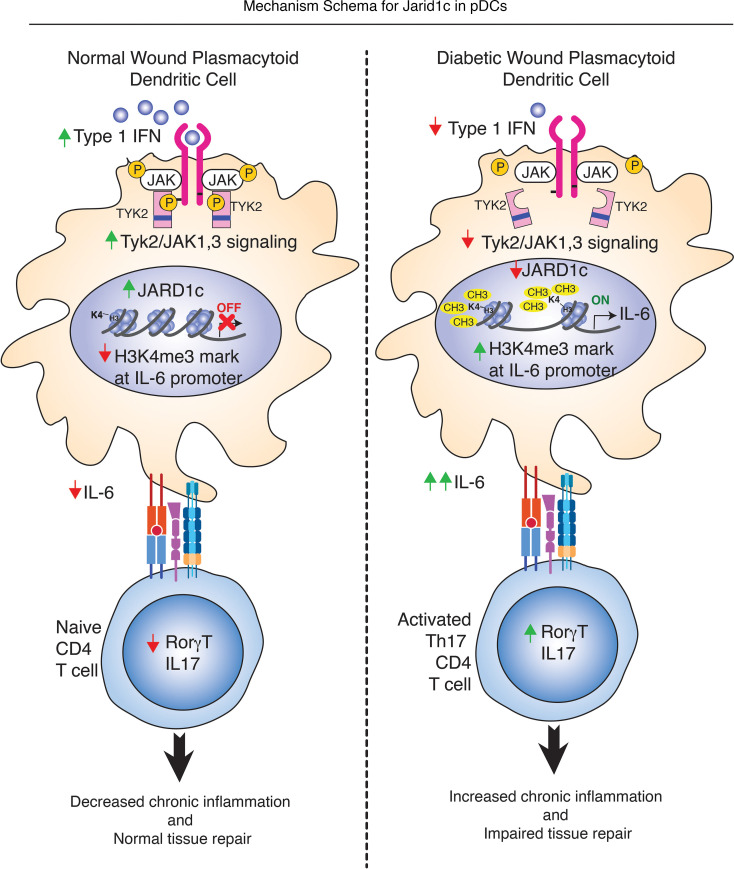

## Introduction

Tissue repair progresses in distinct but overlapping stages, beginning with acute inflammation and concluding with fibrotic scar formation and remodeling (1–4). Antigen-presenting cells (APCs) link the early, innate immune response to the chronic, adaptive immune response in tissue repair (5). Plasmacytoid dendritic cells (pDCs) are circulating, professional APCs that arrive in tissue early after injury and interact with various cells, including naive CD4^+^ T cells, to prime them toward reparative phenotypes following injury (5–8). Originally discovered in humans as a small subset of blood leukocytes responsible for the secretion of type I interferon (IFN-I) in response to infection, pDCs have since been found to play key roles in initiating inflammation (5, 8) and are known as necessary regulators of homeostasis of cytotoxic effector CD8^+^ and regulatory CD4^+^ T cells (6, 7), effectively linking the acute-phase, innate immune response to the chronic, adaptive one. Under steady-state conditions, pDCs circulate in the bloodstream and secondary lymphoid organs and have a limited presence in skin (9, 10). Following injury, pDCs are recruited to wounds early, where they are short-lived, and serve to recognize pathogen and self-nucleic acids to activate secretion of IFN-I and proinflammatory cytokines that recruit other cells and initiate tissue repair (11). In chronic inflammatory diseases of the skin, like psoriasis, pDCs have been shown to chronically produce IFN-α/β, which triggers the expansion of autoimmune T cells and epidermal hyperproliferation (12).

Interleukin-17A–secreting (IL-17A–secreting) CD4^+^ T cells (Th17 cells) are a distinct subset of Th cells that contribute to tissue inflammation and whose lineage expansion is regulated by the expression of IL-6 and TGF-β (13–16). Overexpression of Th17 CD4^+^ T cells is pathogenic, and these cells have been implicated in the chronic inflammation seen in neurological disorders, leukemia, and autoimmune diseases such as rheumatoid arthritis, asthma, lupus, and allograft rejection (17–21). The factors that regulate the expansion of these cells in wound tissue following injury are presently unknown. Understanding these factors is necessary for the development of effective therapeutics for pathologic wound healing.

In conditions associated with nonhealing wounds, such as type 2 diabetes (T2D), wounds fail to progress through the normal stages of repair. Our laboratory and others have shown that wound tissue in T2D exhibits a disproportionately late increase in inflammatory macrophages and other cells that serves to promote chronic inflammation (1, 22, 23). While there are many factors and cell types that lead to this increase in chronic, low-grade inflammation in the setting of T2D, the role of the adaptive immune system in this process has been less studied. There is evidence to suggest that the adaptive immune response in T2D wounds is mediated, at least in part, by pro-inflammatory Th17 cells (24–26); however, the mechanisms responsible for this remain unclear. In particular, the role of T2D wound pDCs in driving this CD4^+^ Th cell regulation is unknown, leading us to explore the interactions between pDCs and naive CD4^+^ T cells in the setting of diabetic wound repair.

Herein, we demonstrate that pDCs in T2D wounds secrete significantly higher amounts of IL-6, a cytokine that has been shown to skew naive CD4^+^ T cells to a Th17 phenotype in the presence of TGF-β (13, 14). Although known to be important for normal wound healing (27), in diabetes IL-6 persists past the acute inflammatory phase and thereby retards progression through the subsequent wound healing phases (28). We found that increased IL-6 expression by diabetic wound pDCs is due to decreased levels of Jumonji AT-rich interactive domain 1C histone demethylase (JARID1C) (also known as KDM5C) at the *Il6* and *Tgfb* promoters, resulting in increased methylation of lysine 4 on histone 3 (H3K4me3), and hence, increased gene transcription. The resulting elevated IL-6 cytokine production by these diabetic pDCs ultimately leads to increased Th17 CD4^+^ cell expansion, consistent with our single-cell RNA-sequencing analysis of human diabetic wounds. We also show that addition of recombinant JARID1C to diabetic wound pDCs leads to decreased IL-6 cytokine production and subsequently decreased Th17 CD4^+^ cell conversion ex vivo and improved wound healing histologically. Finally, we show that JARID1C production is dependent on upstream IFN-I signaling via the tyrosine kinase-2/Janus kinase-1 (TYK2/JAK1) signaling pathway. Together, these data suggest a role for diabetic wound pDCs in orchestrating the maladaptive immune response in diabetic wounds, likely contributing to the impaired tissue repair observed in these wounds.

## Results

### Wound pDCs in diabetic tissue injury produce IL-6 and TGF-β.

pDCs play a key role in normal cutaneous tissue repair by secreting IFN-I, promoting the acute inflammatory response, and priming reparative Th cell expansion (10, 11, 29). It is known that in normal wound healing, depletion of pDCs significantly impairs the acute inflammatory cytokine response and delays reepithelialization of cutaneous wounds (11). Under physiologic conditions, the acute inflammatory stage is short-lived, and the wound progresses through the normal stages of healing, whereas in the diabetic state, the initial inflammatory response to tissue injury is prolonged, leading to failure to progress through the healing cascade and ultimately poor healing. Thus, acute IL-6–mediated inflammation is necessary to initiate wound healing. Efforts by our lab and others to globally inhibit IL-6 in diabetic mice led to diminished healing, suggesting that the initial IL-6–mediated inflammatory response is necessary for wound healing. The role played by wound pDCs in maintaining the chronic inflammatory state in T2D is unknown.

**Figure 1:**
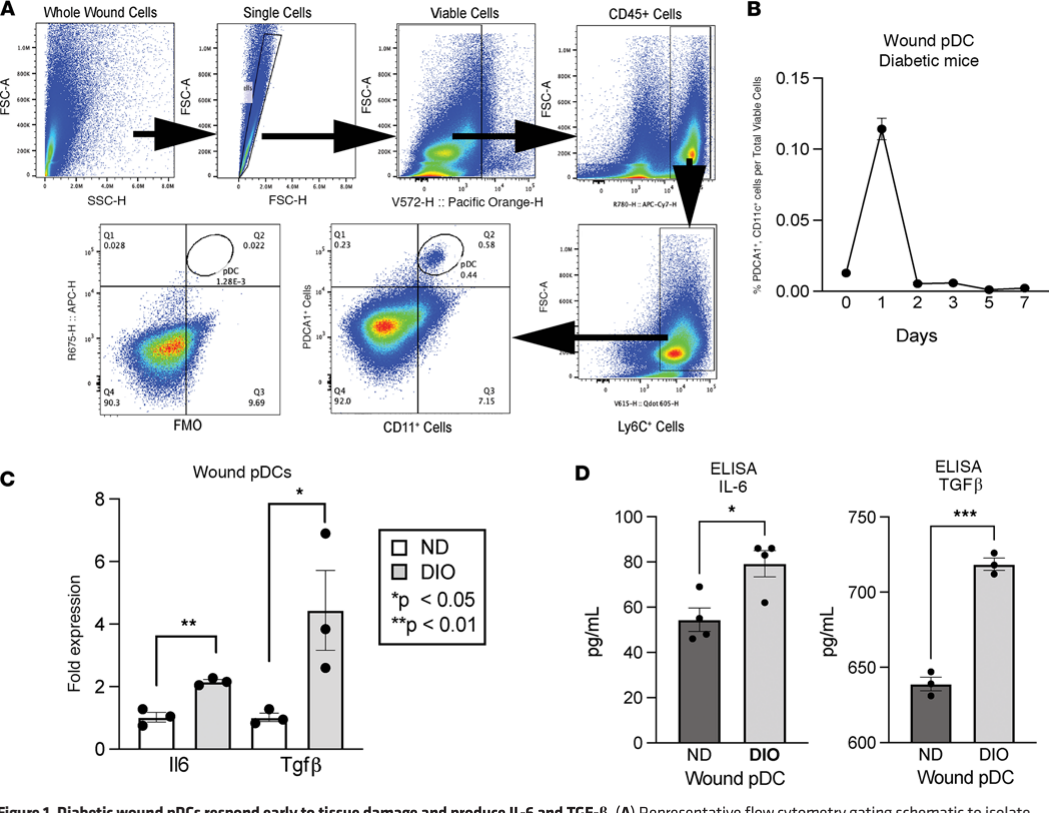
Diabetic wound pDCs respond early to tissue damage and produce IL-6 and TGF-β. (**A**) Representative flow cytometry gating schematic to isolate pDCs (CD45^+^, Ly6C^+^, CD11c^+^, PDCA1^+^) from murine diabetic wounds. (**B**) Kinetic plot of diabetic wound pDCs from 6 mm wounds over time (*N* = 5/group, pooled and repeated in triplicate) determined by flow cytometry. (**C**) mRNA fold expression of *Il6* and *Tgfb* in isolated wound pDCs from diabetic and nondiabetic control mice. (*N* = 3–5/group, pooled, repeated in triplicate.) (**D**) Protein expression of IL-6 and TGF-β in isolated wound pDCs from diabetic and nondiabetic control mice. (*N* = 3–5/group, pooled, repeated in triplicate.) Wound pDCs were isolated using EasySep magnetic bead pDC negative-selection kit, according to manufacturer instructions. *P < 0.05, **P < 0.01, ***P < 0.001. Data are presented as the mean ± SEM and were analyzed using 2-tailed Student’s *t* test.

To examine the role of wound pDCs in diabetic tissue following injury, we first studied the kinetics of pDC infiltration into diabetic tissue following injury. We generated a murine model of obesity and prediabetes by administering a high-fat diet (HFD) chow (60% carbohydrates versus 12% in normal chow) to wild-type mice on a C57BL/6 background for 12–20 weeks. These diet-induced obesity (DIO) mice exhibit insulin resistance and impaired glucose tolerance (30–34). Acute wounds were then created using 6 mm punch biopsies on the dorsum of mice and their normal diet (ND) controls as previously described (35, 36), and wound tissue was harvested and analyzed on days 0–10. Wounds were processed and flow cytometry was performed where pDCs were identified (CD45^+^, Ly6C^+^, CD11c^+^, plasmacytoid dendritic cell antigen-1–positive [PDCA1^+^]) as previously detailed (37, 38) (Figure 1A and Supplemental Figure 4). We found that diabetic wound pDCs are present early in tissue repair (days 0–2) and are absent from the wound bed 48 hours after the initial insult (Figure 1B), which is similar to pDC kinetics in other inflammatory conditions (8). Interestingly, there were fewer pDCs in the diabetic wound compared with the nondiabetic state (Supplemental Figure 7; supplemental material available online with this article; https://doi.org/10.1172/jci.insight.172959DS1). To examine the functional output of the diabetic pDCs, we isolated pDCs by flow cytometry sorting at 24 hours after injury, a time at which they peak in diabetic tissues. We examined an array of cytokines known to be produced in wound tissue by pDCs (6, 39) and found that DIO wound pDCs demonstrated significantly increased *Il6* and *Tgfb* gene expression (Figure 1C) and protein levels (Figure 1D) when compared with normal wound pDCs. These findings demonstrate functional differences in cytokine production by DIO wound pDCs as compared with control, ND wound pDCs.

### Histone demethylase JARID1C regulates IL-6 production in diabetic wound pDCs.

**Figure 2:**
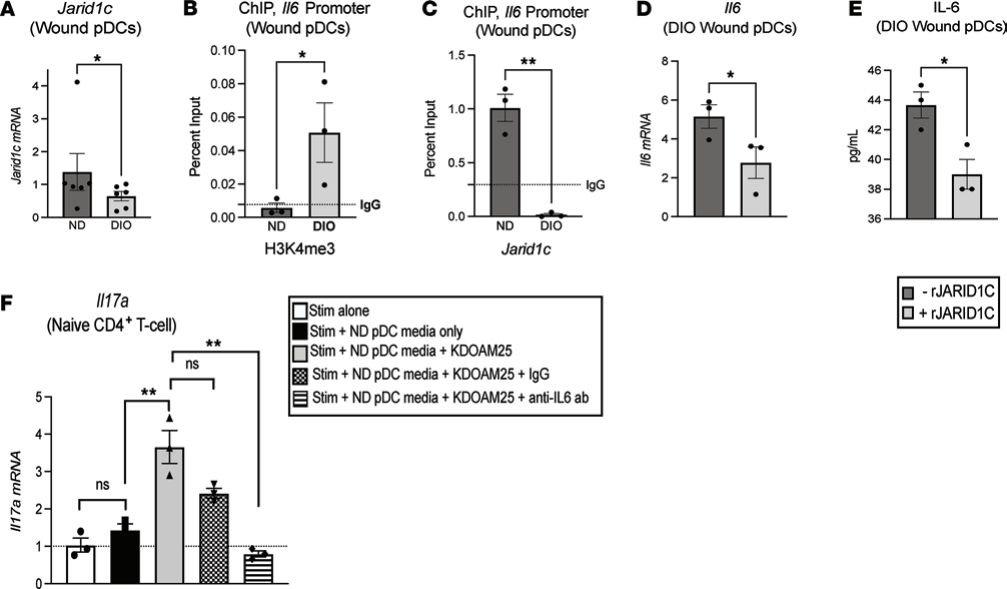
Histone demethylase JARID1C regulates IL-6 in diabetic wound pDCs and affects T cell *Il17a* expression. (**A**) *Jarid1c* expression in ND and DIO wound pDCs. (*N* = 6/group, pooled, repeated in triplicate.) (**B**) ChIP of the H3K4me3 mark on the *Il6* promoter in wound pDCs. (*N* = 6/group, pooled, repeated in triplicate.) (**C**) ChIP of *Jarid1c* at the *Il6* promoter in wound pDCs. (*N* = 3–5/group, pooled, repeated in triplicate.) (**D**) Fold expression of *Il6* in diabetic wound pDCs with and without recombinant JARID1C (rJARID1C, 10 nM, 24 hours; *N* = 3–5/group, pooled, repeated in triplicate). (**E**) Protein expression determined by ELISA of IL-6 in diabetic wound pDCs with and without rJARID1C (10 nM, 24 hours; *N* = 3–5/group, pooled, repeated in triplicate). (**F**) Fold expression of stimulated CD4^+^ T cell *Il17a* following incubation with supernatant from nondiabetic wound pDCs with and without JARID1 inhibition for 24 hours (KDOAM25, 500 nM; *N* = 3/group, pooled, repeated in triplicate) in the presence and absence of anti–IL-6 antibody. For each experiment, wound pDCs were harvested on day 1 after injury and isolated using EasySep pDC negative-selection magnetic bead kit. *P < 0.05, **P < 0.01. All data are presented as mean ± SEM. Data in **F** were statistically analyzed using 1-way ANOVA with Holm-Šidák multiple-comparison test. For all other panels, data were analyzed using 2-tailed Student’s *t* test once normality was assessed.

Studies from our laboratory and others have demonstrated that IL-6 is typically elevated in diabetic wound tissue (28, 40–42). Since IL-6 has been shown to be a primary cytokine in the differentiation of CD4^+^ Th cell phenotypes, particularly in Th17 cell production (13, 14, 43), we focused our efforts on identifying the factors contributing to the increased production of IL-6 in diabetic wound pDCs. To begin, a histone expression profile was obtained by performing a histone superarray on ND and DIO wound pDCs sorted on day 1 after injury. DIO wound pDCs were noted to have significantly decreased JARID1C as compared with ND controls (also referred to as KDM5C; Supplemental Figure 1). JARID1C is a histone demethylase that removes methyl groups from H3K4me3 at gene promoters, thereby decreasing the activating H3K4me3 mark and causing chromatin conformational changes associated with decreased gene transcription. In order to examine if other chromatin-modifying enzymes (CMEs) that regulate H3K4me3 were involved, additional studies were performed to verify that other CMEs traditionally involved in activating the H3K4me3 mark (namely *Mll1*, *Jarid1a*, *Jarid1b*, *Jarid1d*) were not involved in pDCs (Supplemental Figure 2). Similarly, other common epigenetic marks, such as H3K9me3, H3K27me3, and histone 3 acetylases that were previously shown by our group and others to be involved in macrophage-mediated wound repair, were not activating at the *Il6* promoter in diabetic wound pDCs (Supplemental Figure 3). We then verified JARID1C was decreased in diabetic wound pDCs as compared with ND pDCs (Figure 2A). The decreased expression of JARID1C in DIO wound pDCs was associated with an increase in the H3K4me3 mark on the *Il6* promoter (Figure 2B) and a decrease in JARID1C on the *Il6* promoter (Figure 2C). To determine the direct role of JARID1C on IL-6 production, DIO wound pDCs were isolated by cell sorting and treated ex vivo with recombinant JARID1C (rJARID1C, 10 nM) for 24 hours. DIO wound pDCs treated with rJARID1C demonstrated decreased *Il6* gene expression and protein levels (Figure 2, D and E) compared with controls.

To determine the effect of JARID1C in pDCs in directing naive CD4^+^ T cell phenotype, nondiabetic wound pDCs were isolated, then treated with and without a JARID1 inhibitor (KDOAM25, 500 nM) for 24 hours, and the cell-free supernatant was subjected to naive CD4^+^ T cells that were stimulated with anti-CD3/anti-CD28 for 48 hours. This study revealed that nondiabetic pDC supernatant alone did not increase naive T cell *Il17a* production. Inhibition of JARID1 by KDOAM25, which simulates the diabetic state, increases naive T cell *Il17a* production. *Il6* inhibition by anti–IL-6 antibody, in addition to JARID1 inhibition, resulted in a significant decrease in naive T cell *Il17a* production compared with IgG control (Figure 2F). Together, these data suggest that JARID1C modulates pDC IL-6 inflammatory cytokine production through an H3K4me3 mechanism and plays a role in driving naive CD4^+^ T cell phenotype in wounds.

### IFN-β regulates Jarid1c expression in diabetic wound pDCs via TYK2/JAK1 signaling.

**Figure 3:**
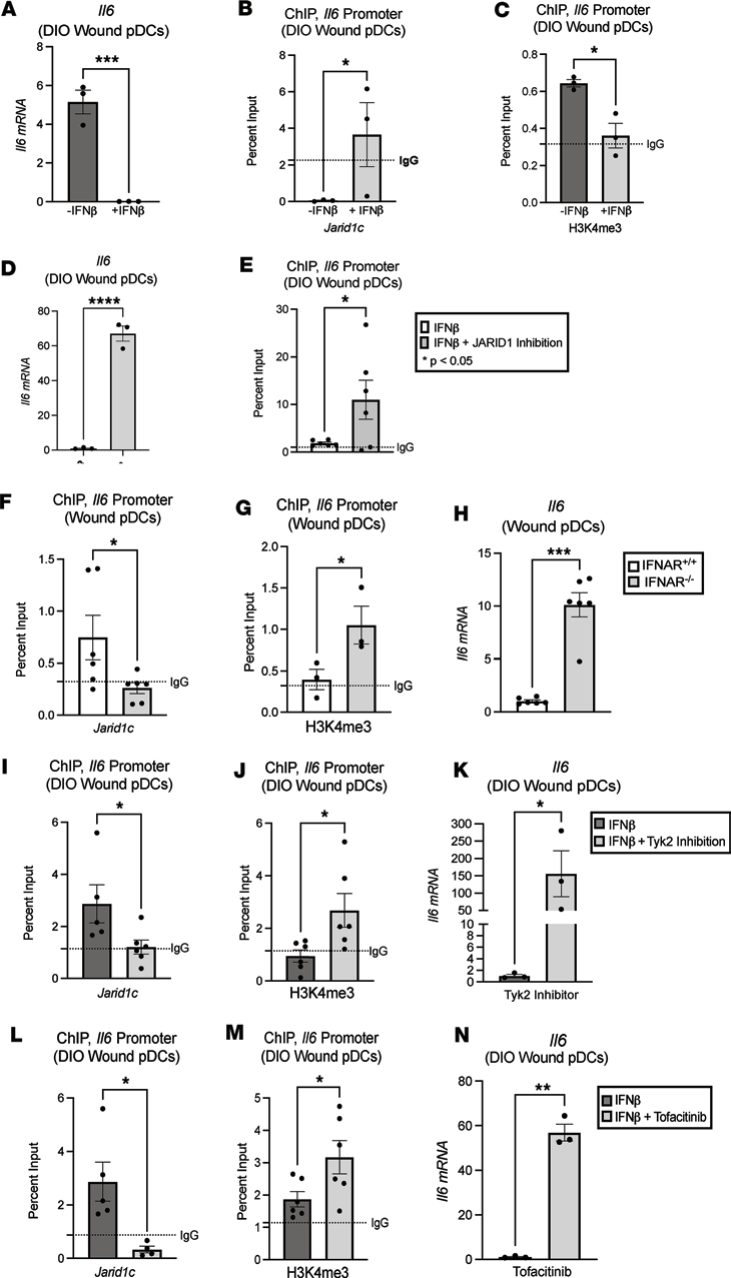
IFN-β regulates JARID1C via TYK2/JAK1,3 signaling. (**A**) *Il6* expression in DIO wound pDCs with and without recombinant IFN-β. (**B**) ChIP analysis of *Jarid1c* on the *Il6* promoter in diabetic wound pDCs with and without IFN-β stimulation. (**C**) ChIP of the H3K4me3 mark on the *Il6* promoter in DIO wound pDCs with and without IFN-β stimulation. (**D**) *Il6* expression in DIO wound pDCs with IFN-β stimulation only with and without JARID1 inhibition. (**E**) ChIP of the H3K4me3 mark on the *Il6* promoter in DIO wound pDCs with IFN-β stimulation, with and without JARID1 inhibition. The graphs are color-coded according to the key, which denotes the cell treatment. (**F**) ChIP of *Jarid1c* at the *Il6* promoter in IFNAR^–/–^ mouse wound pDCs compared with their age-matched littermate controls. (**G**) ChIP of the H3K4me3 mark on the *Il6* promoter in IFNAR^–/–^ mouse wound pDCs, compared with their age-matched littermate controls. (**H**) *Il6* expression in wound pDCs from IFNAR^–/–^ mice and their age-matched littermate controls. (**I**) ChIP of *Jarid1c* at the *Il6* promoter in DIO mouse wound pDCs with a TYK2 inhibitor. (**J**) ChIP of the H3K4me3 mark at the *Il6* promoter in DIO mouse wound pDCs with TYK2 inhibition. (**K**) *Il6* expression in DIO wound pDCs following TYK2 inhibition. (**L**) ChIP of *Jarid1c* on the *Il6* promoter in DIO wound PDCs following JAK1,3 inhibition. (**M**) ChIP of the H3K4me3 mark on the *Il6* promoter in DIO mouse wound pDCs following JAK1,3 inhibition. (**N**) *Il6* expression in DIO wound pDCs after JAK1,3 inhibition. *P < 0.05, **P < 0.01, ***P < 0.001. All experiments were conducted with *N* = 3–6 mice/group, pooled and repeated in triplicate. Murine wound pDCs were harvested on day 1 after injury and isolated using EasySep pDC negative-selection kit. Data are presented as the mean ± SEM. Data were first analyzed for normal distribution, and if data passed the normality test, 2-tailed Student’s *t* test was used.

We, and other laboratories, have shown that IFN-Is are necessary for tissue repair; however, IFN-I expression remains significantly decreased in diabetic wounds (1, 44, 45). We therefore examined if the decreased IFN-I levels in diabetic wounds was responsible, in part, for the diminished JARID1C expression observed in diabetic pDCs. The TYK2/JAK1 signaling cascade is implicated in dendritic cell migration and activation in inflammatory disease processes, such as psoriasis and type 1 diabetes (46, 47). In addition, IFN-I, via the TYK2/JAK1 signaling cascade, is known to regulate IL-6 expression (48, 49). Given the important role of IL-6 to the pDC priming of T cells, we examined if the IFN-I/TYK2/JAK1 signaling pathway controlled JARID1C in diabetic wound pDCs. To examine this, 6 mm punch biopsy wounds were created on DIO mice, and after 24 hours, wound pDCs were isolated and treated ex vivo with or without IFN-β (100 U/mL; 8.5 ng/mL) for 24 hours. DIO wound pDCs treated with IFN-β displayed decreased *Il6* expression as compared with untreated DIO wound pDCs (Figure 3A). Notably, IFN-β administration resulted in increased *Jarid1c* as well as decreased H3K4me3 at the *Il6* promoter as determined by ChIP (Figure 3, B and C). When treated with IFN-β in the presence of a JARID1 inhibitor (KDOAM25, 500 nM), DIO wound pDCs demonstrated increased *Il6* expression (Figure 3D) and a concomitant increase in the H3K4me3 activating mark at the *Il6* promoter as demonstrated by ChIP analysis (Figure 3E). To mimic the diabetic state, we obtained nondiabetic mice globally deficient in the IFN-α,β receptor (IFNaR^–/–^ mice), created 6 mm punch biopsy wounds, and harvested their wound pDCs 24 hours later. Compared with controls, the wound pDCs from the IFNaR^–/–^ mice displayed increased *Il6* mRNA expression (Figure 3F), decreased *Jarid1c*, and an increased H3K4me3 activating mark at the *Il6* promoter by ChIP (Figure 3, G and H), mimicking the diabetic state.

Finally, we then sought to elucidate the role of TYK2/JAK1 on *Jarid1c* and downstream *Il6* production in DIO wound pDCs. DIO mice were wounded with a 6 mm punch biopsy, and wound pDCs were harvested 24 hours later. These DIO wound pDCs were treated ex vivo with IFN-β (100 U/mL; 8.5 ng/mL) with and without TYK2 inhibition (deucracitinib, 500 nM) for 24 hours. Compared with control, the pDCs treated with TYK2 inhibitor exhibited decreased *Jarid1c* (Figure 3I), increased H3K4me3 activating mark (Figure 3J) at the *Il6* promoter by ChIP, and increased *Il6* expression (Figure 3K). Similarly, isolated wound pDCs were treated ex vivo with IFN-β with and without JAK1,3 inhibitor (tofacitinib, 50 nM) for 24 hours. Compared with controls, the pDCs treated with JAK1,3 inhibition exhibited decreased *Jarid1c* (Figure 3L), increased H3K4me3 activating mark (Figure 3M) at the *Il6* promoter by ChIP, and increased *Il6* mRNA expression (Figure 3N).

### Diabetic pDCs promote Th17 CD4^+^ cell expansion.

**Figure 4:**
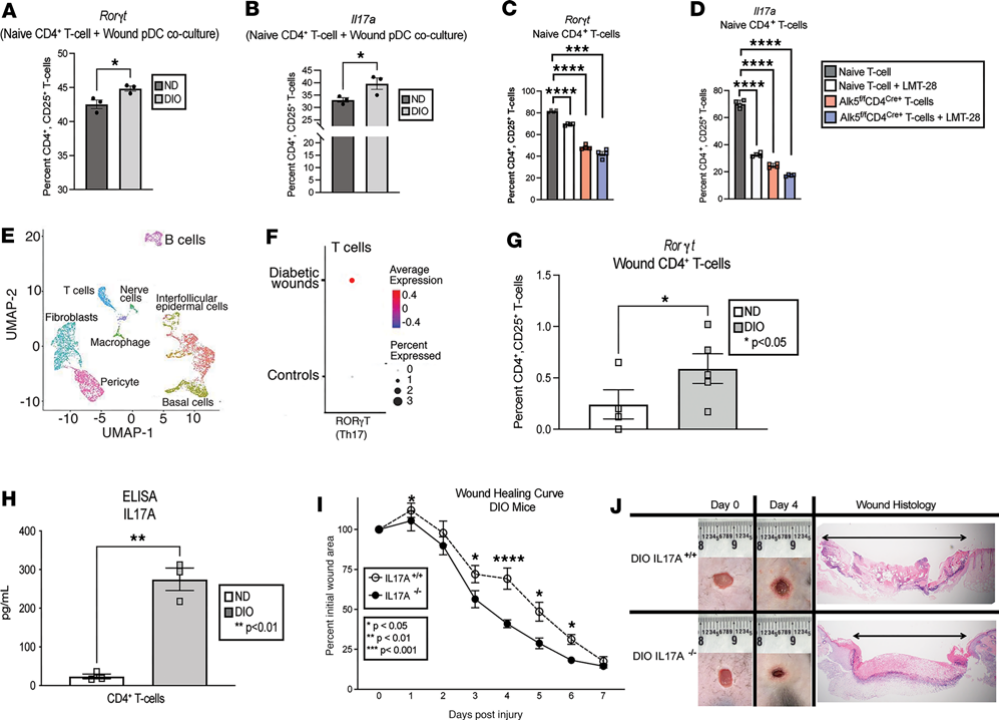
Diabetic wound pDCs promote T cell differentiation toward Th17 phenotype. (**A**) Flow cytometry analysis of RORγt expression in naive CD4^+^ T cells cocultured for 48–72 hours with DIO and ND wound pDCs. (**B**) Flow cytometry analysis of IL-17A in CD4^+^ T cells cocultured for 48–72 hours with DIO and ND wound pDCs. (**C**) RORγt expression in CD4^+^ T cells after 48- to 72-hour coculture with DIO wound pDCs under several conditions — naive CD4^+^ T cells (control), naive CD4^+^ T cells with IL-6 preinhibition (LMT-28, 200 nM, 1 hour), TGF-β receptor–deficient naive CD4^+^ T cells (Alk5^fl/fl^CD4^Cre+^ T cells), and Alk5^fl/fl^CD4^Cre+^ CD4^+^ T cells with IL-6 receptor preinhibition (LMT-28, 200 nM, 1 hour). (**D**) IL-17A expression in CD4^+^ T cells after 48- to 72-hour coculture with DIO wound pDCs under various conditions, as detailed in **C**. For these coculture experiments, each mouse received 3–4 wounds, and *N* = 3–5 mice/group, pooled and repeated in triplicate. (**E**) Cluster uniform manifold approximation and projection (UMAP) of scRNA-Seq from human T2D and non-T2D wounds showed 10 unique cell clusters (representative). (**F**) scRNA-Seq of human wound T-cell population demonstrating RORγt expression in T2D versus non-T2D controls (*N* = 42). Dot size corresponds to proportion of cells within the group expressing RORγt, while dot color corresponds to expression level. (**G**) Intracellular flow cytometry quantifying intracellular RORγt in ND and DIO wound CD4^+^ T cells. (*N* = 3–5 mice/group, pooled and repeated in triplicate.) (**H**) Protein expression by ELISA of IL-17A in ND versus DIO wound CD4^+^ T cells (*N* = 5/group, pooled and repeated in triplicate; day 5 wounds). (**I**) Wound healing curve in global knockout, diabetic IL17A^–/–^ mice compared with age-matched, littermate controls (*N* = 3/group, pooled and repeated in triplicate). (**J**) Representative wound healing images and histology. Data are presented as the mean ± SEM. For **C** and **D**, data were analyzed using 1-way ANOVA with Holm-Šídák multiple-comparison test. Data for **I** were analyzed using 2-way repeated measures ANOVA. For all other panels, data were analyzed using 2-tailed Student’s *t* test once normality was assessed.

Given that IL-6 and TGF-β are well established to work in tandem to influence Th17 CD4^+^ cell expansion (13, 14, 50, 51), we examined if these cytokines produced by diabetic wound pDCs can influence naive CD4^+^ T cell phenotypes. We isolated wound pDCs from ND and DIO mice as well as naive CD4^+^ T cells from the spleens of nondiabetic mice. The pDCs were incubated in coculture with the naive T cells at a ratio of 1:10 (pDC/T cell) for 48–72 hours before analysis with flow cytometry. Intracellular flow cytometry revealed that naive CD4^+^ T cells cocultured with DIO wound pDCs exhibited increased RAR-related orphan nuclear receptor (RORγt) transcription factor, a transcription factor distinctly expressed in CD4^+^ Th17 cells (52). Additionally, they also had increased IL-17A cytokine expression (Figure 4, A and B) when compared with CD4^+^ T cells cocultured with pDCs from their nondiabetic controls. This suggests that naive CD4^+^ T cells are skewed toward the inflammatory Th17 CD4^+^ cell phenotype by DIO wound pDCs ex vivo. To further elucidate the role of pDCs in directing T cell lineage, pDCs from DIO murine wounds were isolated and cocultured under the following conditions: a) naive CD4^+^ T cells (control), b) naive CD4^+^ T cells pretreated with an IL-6 receptor inhibitor (LMT-28, 200 nM, 1 hour) before pDC addition, c) naive CD4^+^ T cells from mice with TGF-β receptor–deficient T cells (Alk5^fl/fl^CD4^Cre+^), and d) naive CD4^+^ T cells from Alk5^fl/fl^CD4^Cre+^ mice with IL-6 receptor inhibition pretreatment. Analysis by flow cytometry revealed a stepwise decrease in intracellular RORγt transcription factor and a decrease in IL-17A cytokine production in CD4^+^ T cells with restriction of IL-6/TGF-β signaling (Figure 4, C and D).

In order to determine if these findings in murine tissue translated to human diabetic wounds, we performed single-cell RNA sequencing (scRNA-Seq) of the CD4^+^ T cell population from human T2D wounds and nondiabetic control wounds that were age and sex matched as previously described by our lab (53). We observed that T2D wounds had increased expression of RORγt (Figure 4, E and F). Intracellular flow cytometry on CD4^+^CD25^+^ T cells isolated from day 5 murine wounds also verified increased Th17 transcription factor (RORγt) expression in the diabetic mouse model (Figure 4G and Supplemental Figure 6) when compared with nondiabetic controls. Wound CD4^+^ T cells isolated by sorting (54) on day 5 after injury from DIO mice also had elevated IL-17A cytokine production by ELISA (Figure 4H). To highlight the detrimental effect of increased IL-17A production in tissue repair, we generated DIO IL-17^–/–^ mice by subjecting global knockout IL-17^–/–^ mice (RRID: IMSR_JAX:033431; The Jackson Laboratory) to an HFD over 12–20 weeks. We then created 6 mm punch biopsy wounds and performed a wound healing time curve comparing DIO IL-17^–/–^ mice and DIO IL-17^+/+^ littermate controls. The DIO IL-17^–/–^ mice exhibited significantly improved wound healing in the later days, when wound CD4^+^ T cells are most abundant (Figure 4, I and J). In sum, diabetic wound CD4^+^ T cells had significantly higher Th17 phenotype predominance, leading to higher IL-17A expression and impaired wound healing. Together, these data suggest that diabetic wound pDCs skew naive CD4^+^ T cells toward a Th17 inflammatory phenotype and that the increased production of IL-6 and TGF-β is partially responsible for this phenomenon.

## Discussion

Diabetes is a chronic inflammatory disease that alters cellular function in the wound and impairs progression through the phases of wound repair. Prior work by our laboratory and others has highlighted the role of T2D in altering macrophage-mediated signaling and activity driving toward a persistent inflammatory phenotype (1, 28, 45, 53, 55–57). Recently our laboratory has highlighted that diabetes also alters structural cells, such as keratinocytes, resulting in impaired keratinocyte/macrophage signaling that promotes inflammation and decreased IFN-I production (22, 23). Our laboratory has also previously demonstrated that IFN-I is critical for wound healing in diabetes. Reconstitution of IFN-I into the diabetic wound bed results in more rapid healing (22, 44). Although innate immune cells (e.g., macrophages) and structural cells (e.g., keratinocytes, fibroblasts) have been well studied in the context of diabetic wound repair, adaptive immune cells, namely CD4^+^ Th cells, have not been well investigated. In the nondiabetic state, following tissue damage, there is marked accumulation of CD4^+^ T cells at the site of injury peaking on day 7 after injury (58). The majority of these T cells are regulatory CD4^+^ T cells (Tregs), characterized by increased expression of CD25, cytotoxic T-lymphocyte antigen-4, and CD278 (59). These Tregs are directly involved in the reparative process, resulting in tissue regeneration and healing, and act to suppress macrophage-mediated inflammation. In diabetes, the balance between Tregs and Th17 cells is skewed to favor Th17. Specifically, several reports show a significant link between diabetes and increased inflammatory CD4^+^ Th17 cell expression (24, 26, 60), and there have been a few studies showing that decreasing CD4^+^ Th17 cell expression in diabetes can lead to decreased pathogenicity (61–63).

Despite this knowledge, the effect of diabetes on the innate immune system’s ability to prime the adaptive immune system has not been robustly explored. Here, diabetic wound pDCs were found to express less histone demethylase JARID1C (KDM5C), allowing for increased transcription of IL-6 (and TGF-β) via an H3K4me3 mechanism. As a result, these diabetic wound pDCs interact with naive CD4^+^ T cells to generate IL-17A–secreting (Th17) CD4^+^ T cells. Although only recently reported, Th17 CD4^+^ cells are potent inflammatory cells, important for sustaining chronic inflammation in various disease states. They require IL-6 and TGF-β for activation from naive T cells (13, 14, 51). We show here that global reduction of IL-17A in diabetic mice improved healing, suggesting this cell-cell interaction and the upstream pathway may be a novel therapeutic target.

The JARID1 family of histone demethylases consists of 4 members (JARID1A–JARID1D) known to demethylate the H3K4me3 activating mark on various gene promoters (64). Collectively, they are implicated in malignancy, cellular proliferation, and most recently in inflammation (65–67). Specifically, JARID1C is primarily known for its role in X-linked chromosomal aberrations (68–70) and has been a target for this pathology, though no JARID1C-specific small molecule inhibitor is currently available. Its role in inflammation has only recently been described within the context of neurological disorders, such as glioblastoma multiforme, where it was shown to be important to the hypoxia-inducible factor 1α (HIF1α) inflammation axis (71, 72). JARID1C has not been implicated in diabetes or tissue repair processes.

Although this study furthers understanding of the mechanisms leading to dysregulated, chronic adaptive inflammation in diabetic wounds, there are limitations to address. First, we acknowledge the lack of Cre-specific transgenic lines for pDCs. This is because of the significant lineage relationship overlap between monocytes/macrophages, neutrophils, and dendritic cells. Second, there are likely other epigenetic enzymes that may regulate pDC function in diabetic wounds. These enzymes likely contribute at various wound repair phases (73–75). Third, we acknowledge that IFN-I is possibly one of several drivers of TYK2/JAK1,3 signaling in wounds. Fourth, we conducted a pilot study to replenish rJARID1C in murine wounds, which revealed improved healing and collagenization by histological evaluation on day 3 after wounding (Supplemental Figure 8). We were unable to perform a full in vivo wound curve utilizing rJARID1C given the prohibitive cost of this newly discovered epigenetic enzyme. Fifth, despite multiple attempts, we were unable to verify increased intracellular staining of IL-6 and TGF-β in DIO wound pDCs by flow cytometry of whole wound lysate, even though we show by mRNA expression and ELISA of isolated DIO wound pDCs that there is increased production of these cytokines in the DIO state. Finally, we did not directly examine the associations between pDCs and resident structural cells in the wound bed. These include smooth muscle cells and fibroblasts. Such studies would further understanding of the factors responsible for chronic T cell–mediated inflammation in diabetic wound healing.

To conclude, our study provides important mechanistic information that pDCs play an integral part in priming Th17 cells by increasing IL-6 production in DIO pDCs via a TYK2/JAK1/JARID1C mechanism. Our results suggest that specific epigenetic targeting of JARID1C in diabetic wound pDCs may be a viable therapeutic strategy to decrease Th17 CD4^+^ cell–mediated inflammation and improve diabetic tissue repair.

## Methods

### Sex as a biological variable.

Sex was not considered as a biological variable in all reported human data. Only males were used in the murine studies, since female C57BL/6 mice do not exhibit the DIO phenotype when placed on an HFD.

### Mice.

Male C57BL/6 mice (RRID: IMSR_JAX:000664) were delivered at 6–7 weeks of age from The Jackson Laboratory, were maintained in breeding pairs in the Unit for Laboratory and Animal Medicine (ULAM) facilities, and were fed a normal chow diet (13.5% kcal fat; LabDiet). IL-17A^–/–^ mice (RRID: IMSR_JAX:033431) are global knockout strain at the IL-17A receptor locus obtained at 6–7 weeks from The Jackson Laboratory and were maintained in breeding pairs in the ULAM facilities. The Alk5^fl/fl^ mice were obtained from Benjamin Levi from the University of Texas Southwestern, Dallas, Texas, USA.

To induce a diabetic phenotype, mice were maintained on a standard HFD (60% kcal saturated fat, 20% proteins, 20% carbohydrate; Research Diets, Inc) for 12–18 weeks to yield the DIO model of T2D (76, 77). HFD-fed (DIO) mice developed obesity and insulin resistance with fasting blood sugars in the mid-200s and elevated insulin levels. All DIO and control animals underwent procedures at 20–32 weeks of age with Institutional Animal Care and Use Committee (IACUC) approval. For these experiments, only male mice were used as female mice do not develop DIO.

### Wound pDC isolation.

Punch biopsy wounds (6 mm) were created on the dorsum of mice and maintained for 24 hours. Wounds were then harvested from the mice postmortem following CO_2_ asphyxiation. Wounds were minced finely with sharp scissors and suspensions enzymatically digested in Liberase (50 mg/mL; MilliporeSigma, catalog 5101020001) and DNase I (20 U/mL; MilliporeSigma catalog 9003-98-9) solutions at 37°C for 30 minutes. RPMI with FBS was then added to stop the reaction, and the wound cells were then gently plunged and filtered through a 100-micron filter (Corning) to result in a single-cell suspension. Murine wound pDCs were isolated by negative selection using EasySep Mouse Plasmacytoid DC magnetic bead isolation kits (catalog: 19764 STEMCELL Technologies) according to manufacturer instructions, which report a purity of 65% to 90% enrichment. This was verified in our hands (Supplemental Figure 5). These isolated cells were then cultured ex vivo for RNA, cDNA, or protein studies.

### Mouse naive CD4^+^ T cell isolation.

Wild-type ND mice were euthanized by CO_2_ asphyxiation and their spleens harvested. The spleens were crushed and filtered through a 100-micron filter, and the suspension was subjected to magnetic activated cell sorting (MACS) using a negative-selection EasySep Mouse CD4^+^ naive T cell isolation kit (STEMCELL Technologies) according to manufacturer instructions. Cells were then cultured ex vivo for RNA, cDNA, protein, or flow cytometry studies. For in vitro studies with pDC supernatant, the naive CD4^+^ T cells were stimulated with 3 μg/mL anti-CD3 antibody (Abcam catalog 135372) and 3 μg/mL anti-CD28 antibody (Abcam catalog 243228). Wells containing naive CD4^+^ T cells were coated with anti-CD3 at 37°C for 2–3 hours. Anti-CD28 was added to cell-free pDC supernatant, and this was used to treat the naive CD4^+^ T cells.

### Wound pDCs and T cell coculture.

Wound pDCs and naive T cells were isolated as previously described and placed in coculture in a 1:10 pDC/T cell ratio for 48–72 hours at 37°C in 5% CO_2_ incubator. The coculture was then prepared for intracellular and extracellular T cell flow cytometry evaluation using appropriate antibodies.

### RNA extraction.

Total RNA extraction was performed with TRIzol (Invitrogen, Thermo Fisher Scientific) using manufacturer’s directions. RNA was extracted using chloroform, isopropanol, and ethanol. Superscript III Reverse transcriptase kits (Thermo Fisher Scientific) were used to synthesize cDNA from extracted RNA. cDNA primers for *Tgfb*, *Il6*, and *Il17a* were purchased from Applied Biosciences, Thermo Fisher Scientific. Reverse transcription PCR (RT-PCR) was conducted with 2× Taqman Fast PCR mix and run on a 7500 Real-Time PCR system (Applied Biosciences, Thermo Fisher Scientific), and data were then reviewed in a relative quantification analysis to the 18S rRNA. All samples were assayed in triplicate. Data were then compiled in Excel (Microsoft) and presented using Prism software (GraphPad v9).

### ChIP assay.

ChIP assay was performed as described previously (78). Briefly, following ex vivo studies, wound pDCs were cross-linked in 1% formaldehyde for 10 minutes at room temperature and pellets stored at –80°C until analysis. Cells were lysed for 10 minutes on ice in SDS lysis buffer supplemented with a protease inhibitor cocktail (MilliporeSigma), syringe passaged, and sonicated to generate 100–300 base pair fragments. Five percent of the total chromatin volume was put aside for the input control. The rest of the chromatin was subsequently incubated with antibodies against *Jarid1c* (Abcam catalog 190180), trimethylated H3K4 (Thermo Fisher Scientific catalog MA5-11199), trimethylated H3K9 (Thermo Fisher Scientific catalog PA5-31910), trimethylated H3K27 (Thermo Fisher Scientific catalog MA5-11198), or rabbit monoclonal IgG (Thermo Fisher Scientific catalog 08-6199) overnight at 4°C. This was followed by addition of Protein A sepharose beads (Thermo Fisher Scientific) for 1 hour at 4°C. The pellet was washed and bound DNA eluted twice for 15 minutes at room temperature for 5 minutes at 65°C at the end of the second elution. The combined eluates were reverse cross-linked for 5 hours at 65°C. Samples were stored at –20°C, followed by a proteinase K digestion for 1 hour at 45°C. DNA was purified using phenol/chloroform/isoamyl alcohol solution. Precipitated DNA was analyzed by quantitative real-time PCR on a TaqMan 7500 sequence detection system (Thermo Fisher Scientific). The following primers were used to amplify DNA in samples: *Il6*: (forward) 5′AGGTTTCCAATCAGCCCCAC3′ and (reverse) 5′GGGCTCCAGAGCAGAATGAG3′.

### Flow cytometry.

Wound cell isolates were collected either directly from wounds or after ex vivo coculture stimulation under varying conditions. Cells were processed for intracellular staining as previously described (45). Briefly, FcRs were blocked with anti-CD16/32 (BioXCell RRID:AB_2687830, CUS-HB-197, 1:200 dilution) for 10 minutes. Monoclonal antibodies for surface staining included anti-CD25–PB (BioLegend; 102021). After surface staining, cells were either washed and processed for surface-only flow cytometry or fixed with 2% formaldehyde and then permeabilized with perm/wash buffer (BD Biosciences, 00-8333-56) for intracellular flow cytometry. Intracellular stains for T cells included cytokines (anti–IL-17A–FITC [BioLegend; catalog 506907], anti–IL-4–APC [BioLegend; catalog 504105], anti–IFN-γ–PE [BioLegend; catalog 505807]) and transcription factors (anti-RORγt–PerCP [R&D Systems, Bio-Techne; catalog IC6006C-025], anti-Gata3–APC [R&D Systems, Bio-Techne; catalog IC63301A], anti-Tbet–PECy7 [BioLegend; catalog 644823], and anti-FoxP3–PE [R&D Systems, Bio-Techne; catalog IC8970P]). Samples were acquired on a 3-laser Novocyte flow cytometer (Acea Biosciences, now Agilent Technologies). Analysis was performed using FlowJo software version 10.7.1 (Tree Star). The usual yield for MAC-sorted pDCs or naive CD4 T cells was 1 × 10^6^ to 2 × 10^6^ cells for 4 wounds per mouse, and we would pool 3 mice into 1 biological sample. These samples were then replicated 3–5 times depending on the experiment, with data reported as the mean and standard error of the mean among the biological samples. All populations were back-gated to verify gating and purity.

### ELISA.

Wound macrophages were MACS-isolated and stimulated in culture for 4 hours in RPMI. After stimulation, cell-free supernatant was collected and analyzed by specific enzyme immunoassay kits for IL-6 and TGF-β (all ELISAs from Cayman Chemical) according to the manufacturer’s instructions.

### Histology.

Whole wounds were excised from mice or humans using a 6 to 8 mm punch biopsy. Wound sections were fixed in 10% formalin overnight before embedding in paraffin. Sections of 5 mM were stained with Masson’s trichrome for evaluation of reepithelialization, granulation, and collagen deposition. Images were quantified on ImageScope software and ImageJ (NIH) at 2× original magnification. Percentage reepithelialization was calculated by measuring distance traveled by epithelial tongues on both sides of wound divided by total distance (79).

### Human wound isolation.

Biopsies from human diabetic wounds (*n* = 4) versus normal skin samples (*n* = 38) were collected from patients recruited from University of Michigan hospitals under IRB approval. The diabetic patient samples for scRNA-Seq had an average age of 60 years, with all patients having diabetes (A1c > 7), hypertension, hyperlipidemia, and coronary artery disease. In the nondiabetic patient samples, the average age was 70 years, with half the patients having hypertension, hyperlipidemia, and coronary artery disease. Wounds were obtained from the specimens using an 8 mm punch biopsy tool and processed for RT-PCR as described for the murine wounds. RNA with RNA integrity number scores of greater than 8 were used, and all values were done with comparison to 28S/18S ratios and other housekeeping genes.

### scRNA-Seq analyses.

Generation of single-cell suspensions for scRNA-Seq was performed in the following manner. Skin was harvested via punch biopsy from diabetic and nondiabetic control patient wounds. Samples were incubated overnight in 0.4% Dispase (Life Technologies, Thermo Fisher Scientific) in HBSS (Gibco, Thermo Fisher Scientific) at 4°C. The epidermis and dermis layers were separated. The epidermis was digested in 0.25% Trypsin-EDTA (Gibco, Thermo Fisher Scientific) with 10 U/mL DNase I (Thermo Fisher Scientific) for 1 hour at 37°C, subsequently quenched with FBS (Atlanta Biologicals), and strained through a 100 μM mesh. The dermis was minced, digested in 0.2% Collagenase II (Life Technologies, Thermo Fisher Scientific) and 0.2% Collagenase V (MilliporeSigma) in plain RPMI medium for 1.5 hours at 37°C, and strained through a 100 μM mesh. Epidermal and dermal cells were combined in a 1:1 ratio for scRNA-Seq by the University of Michigan Advanced Genomics Core on the 10x Genomics Chromium System. Libraries were sequenced on the Illumina NovaSeq 6000 sequencer. NovaSeq was used as the sequencing platform to generate 151 bp paired-end reads. We conducted adapter trimming and quality control procedures as described previously (80). The reads were then mapped using STAR (81) to build human GRCh37, and gene expression levels were quantified and normalized by HTSeq (82) and DESeq2 (83), respectively. Negative binomial models in DESeq2 were used to conduct differential expression analysis. To increase the sample size of the control samples, we used the skin biopsies obtained from our previous study (19). For scRNA-Seq data, data processing, including quality control, read alignment, and gene quantification, was conducted using the 10x Genomics Cell Ranger software. Seurat was then used for normalization, data integration, and clustering analysis as previously described (84). All clustered cells were mapped to corresponding cell types by matching cell cluster gene signatures with putative cell type–specific markers.

### Statistics.

GraphPad Prism software (RRID:SCR_002798) version 9.2.0 was used to analyze the data. Except where explicitly stated, all data were analyzed for normal distribution, and then statistical significance between multiple groups was obtained using Student’s 2-tailed *t* tests. All *P* values less than or equal to 0.05 were considered significant.

### Study approval.

All experiments using human samples were approved by the IRB at the University of Michigan (IRB HUM00098915) and were conducted in accordance with the principles in the Declaration of Helsinki. All mice used were on a C57BL/6 background. Mice were housed at the University of Michigan Biomedical Sciences and Research Building in the ULAM, a pathogen-free animal facility. Mouse experiments were conducted with approval from our IACUC (Protocol no. PRO00009811), and all regulatory and safety standards were strictly adhered to.

### Data availability.

All underlying source data are available in an Excel file labeled “Supporting Data Values” accompanying the manuscript. For bulk RNA-Seq and scRNA-Seq data accession, numbers include GSE154556 and GSE154557 (National Center for Biotechnology Gene Expression Omnibus).

## Author contributions

COA designed the research studies, performed the experiments, acquired the data, analyzed the data, and wrote and edited the manuscript. RW and LCT acquired and analyzed the scRNA-Seq data. SJW, JYM, WJM, ADJ, SBS, FMD, ATO, ECB, KDM, and TMB aided in experimental design and approach, provided reagents, performed experiments, and edited the manuscript. SLK, BBM, and KAG aided in experimental design and data analysis, and edited the manuscript.

## Supplementary Material

Supplemental data

Supporting data values

## Version 1 06/24/2024

Electronic publication
