# Supplementary material for: Histone demethylase JARID1C/KDM5C regulates Th17 cells by increasing IL-6 expression in diabetic plasmacytoid dendritic cells
Source: JCI Insight. 2024 Jun 24;9(12):e172959. doi: 10.1172/jci.insight.172959 (PMC11383169; doi:10.1172/jci.insight.172959)
Supplement: Supplemental data [file jciinsight-9-172959-s112.pdf]

SUPPLEMENTAL FIGURES & LEGENDS

Sample ID	Sample Name	Group
1	cont	Control Group
2	DIO	Group 1

Genes Over-Expressed in Group 1 vs. Control Group

Position	Gene Symbol	Fold Regulation	Comments	RT² qPCR Assay Catalog #
A08	Ciita	5.46		PPM03368F

Genes Under-Expressed in Group 1 vs. Control Group

	Position	Gene Symbol	Fold Regulation	Comments	RT² qPCR Assay Catalog #
	A01	Ash1l	-2.65		PPM31189C
	A02	Atf2	-14.71		PPM03036C
	B09	Hdac10	-2.11		PPM41767B
	C05	Hdac8	-4.05		PPM30920A
**	D02	Kdm5c	-3.73		PPM31522A
	F04	Setd1a	-2.72		PPM41827C
	F08	Setd4	-2.11		PPM25607B
	G01	Setdb1	-2.13		PPM32502A
	G09	Usp16	-2.01	A	PPM31724A
	G10	Usp21	-2.31	A	PPM42218C

Supplemental Fig 1. Histone superarray in wound pDCs. Wound pDCs from DIO mice and controls were subjected to a histone array studies (Abcam) detecting chromatin modifying enzyme changes, including JARID1C/KDM5C depletion (asterisks) in DIO wound pDCs.

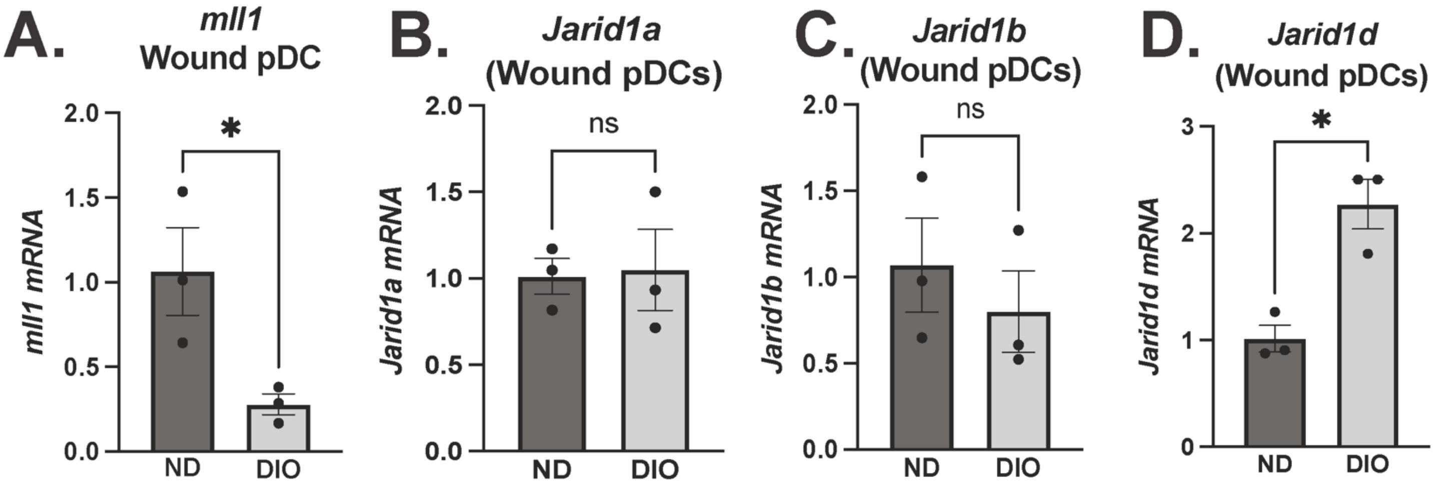

Supplemental Fig 2. Evaluation of common H3K4me3 modifying enzymes on wound pDCs. A) *mll1* gene expression in DIO wound pDCs. B) *Jarid1a* gene expression in DIO wound pDCs. C) *Jarid1b* gene expression in DIO wound pDCs. D) *Jarid1d* gene expression in DIO wound pDCs. For all experiments, N=4 mice/group, pooled, run as triplicates. *$p < 0.05$. Data are presented as the mean ± SEM. Significance determined by paired student's t-test.

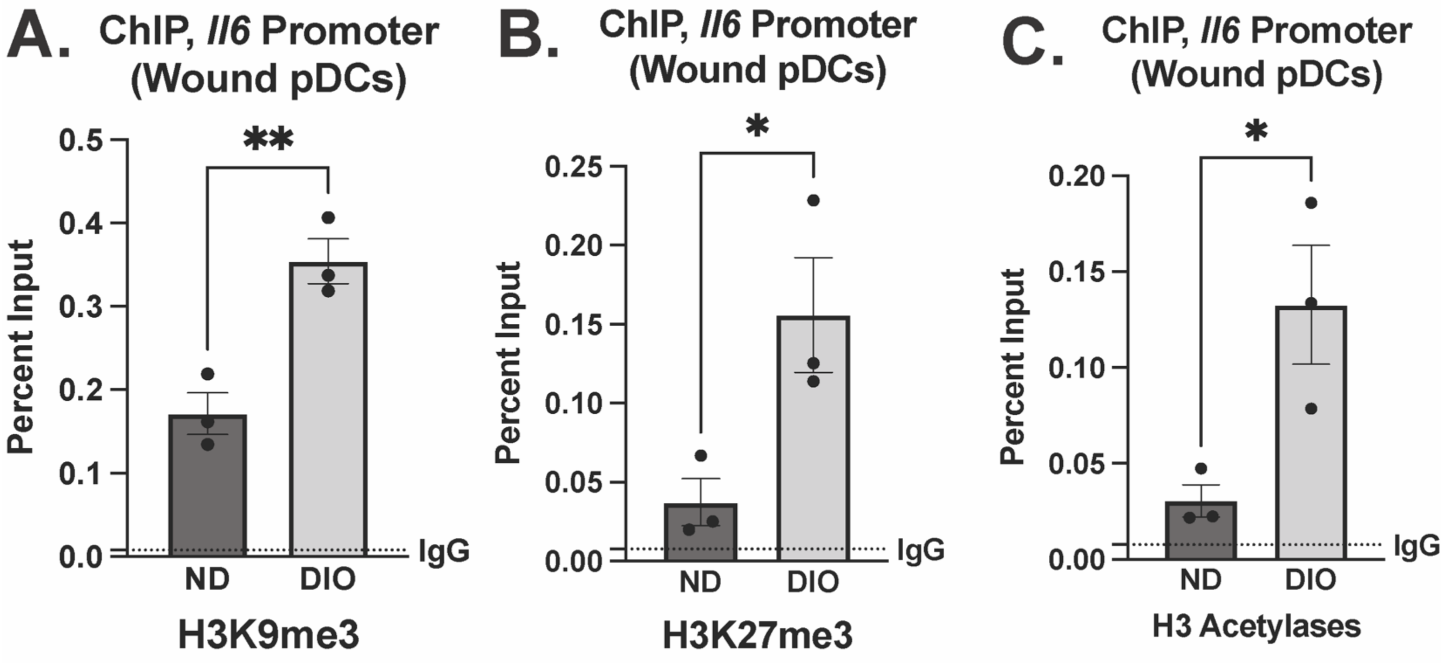

Supplemental Fig 3. Screen of other H3 marks. A) ChIP analysis of H3K9me3 on the *Il6* promoter in ND and DIO wound pDCs. B) ChIP analysis of H3K27me3 mark on the *Il6* promoter in ND and DIO wound pDCs. C) ChIP analysis of H3 acetylase mark on the *Il6* promoter in ND and DIO wound pDCs. For all experiments, N=3 mice/group, pooled, run as technical triplicates. *$p < 0.05$, **$p < 0.01$, Data are presented as the mean ± SEM. Significance determined by paired student's t-test.

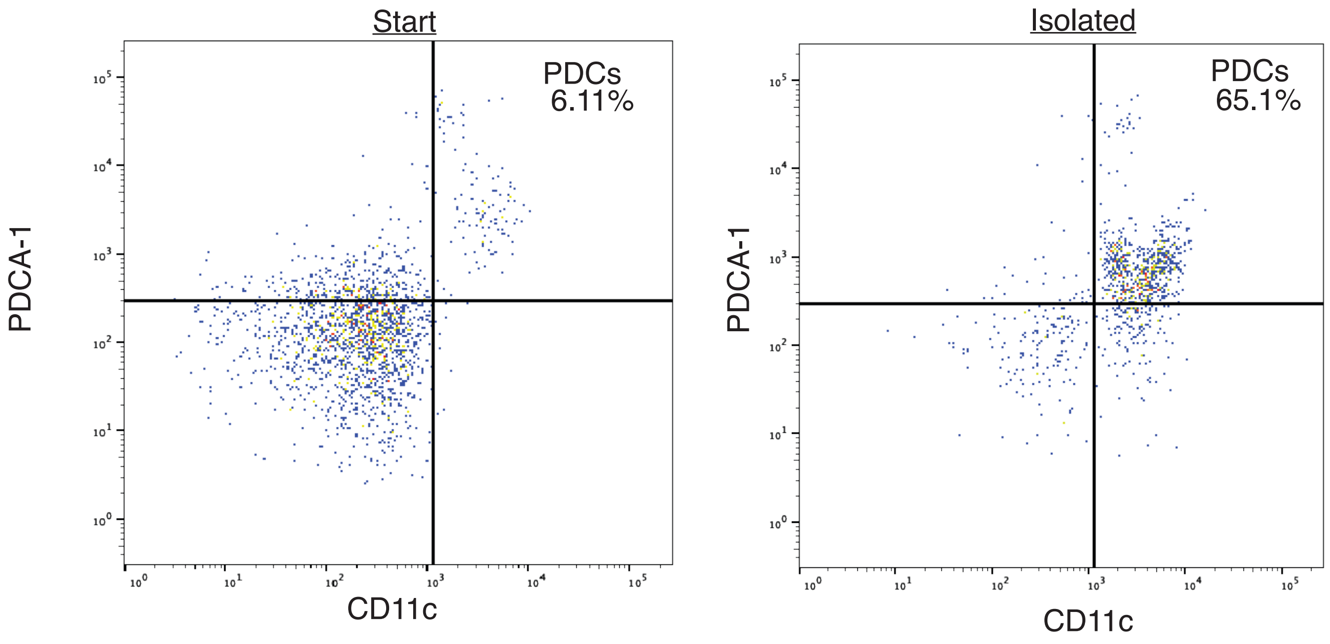

Supplemental Fig 4. pDC Enrichment. Sample flow cytometry showing pDC enrichment following isolation from wounds with EasySep^{TM} Mouse Plasmacytoid DC magnetic bead isolation kits (catalog #:19764) according to manufacturer instructions.

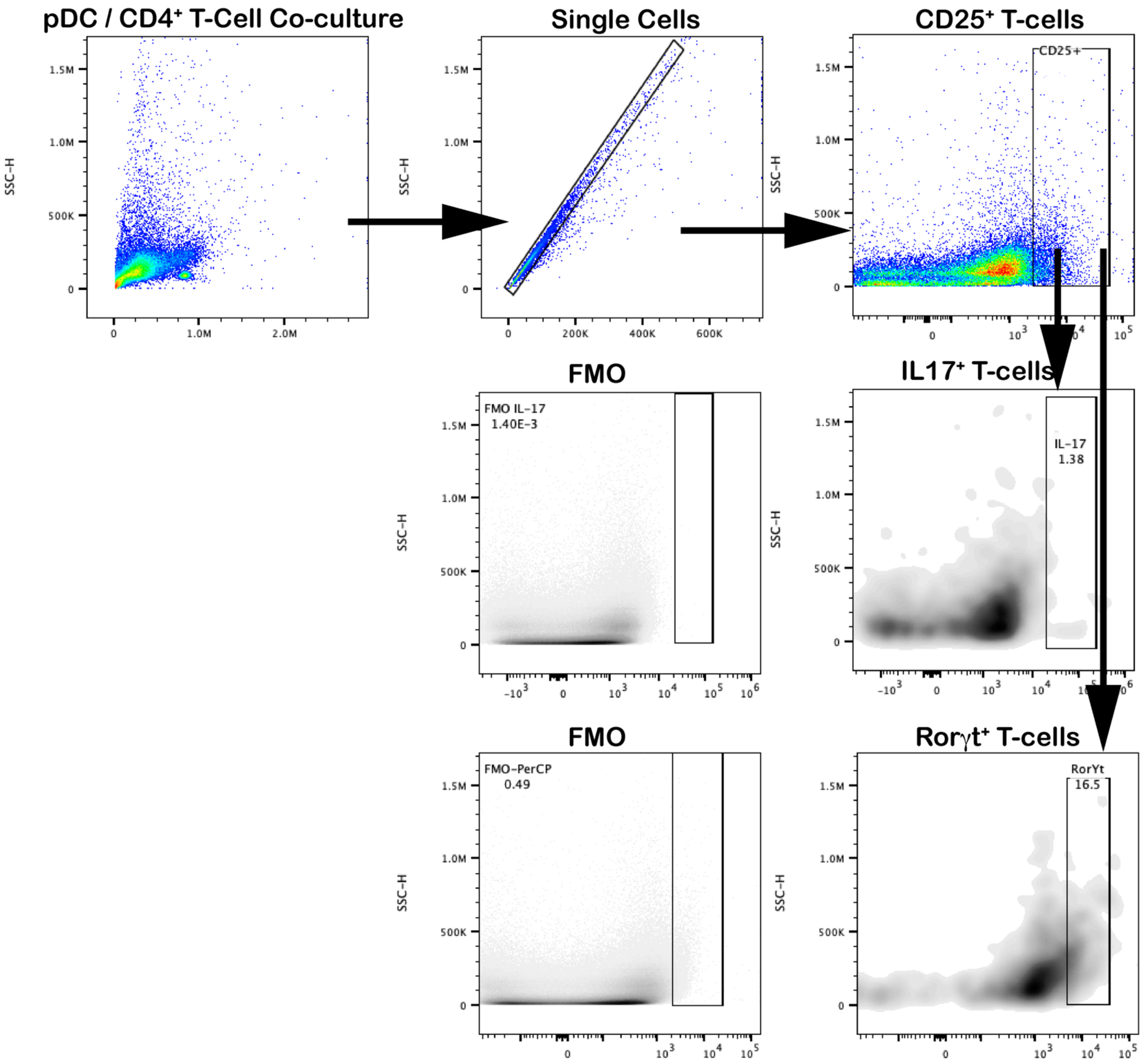

Supplemental Fig 5. Naïve CD4 T-cell gating strategy following co-culture with pDC. Sample flow cytometry gating for Rorγt and IL17 in CD4+, CD25+ T-cells following co-culture experiments with either ND or DIO pDCs (72hr incubation; 10:1 T-cell:pDC ratio). T-cell were isolated using EasySep™ Mouse Naïve CD4+ T-cell magnetic bead isolation kit (catalog #: 19765) and pDC were isolated using EasySepTM Plasmacytoid DC magnetic bead isolation kits (catalog #:19764) according to manufacturer instructions.

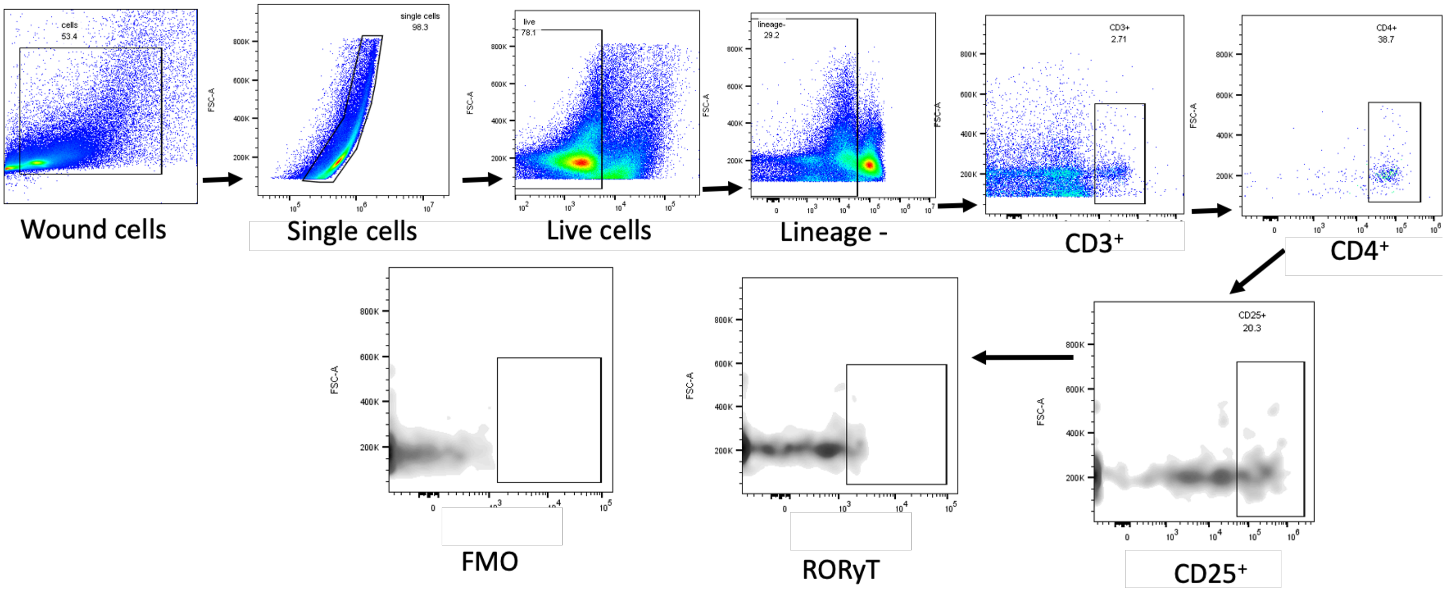

Supplemental Fig 6. Wound T-cell gating strategy. Example flow cytometry gating for Rorγt in CD4$^+$, CD25$^+$ T-cells derived directly from murine ND or DIO wounds on day 5 after wounding. Whole wound cells were harvested, minced, and filtered (100 μm filter) and the filtrate subjected to flow cytometry after staining for relevant T-cell extracellular and intracellular antibodies (Rorγt).

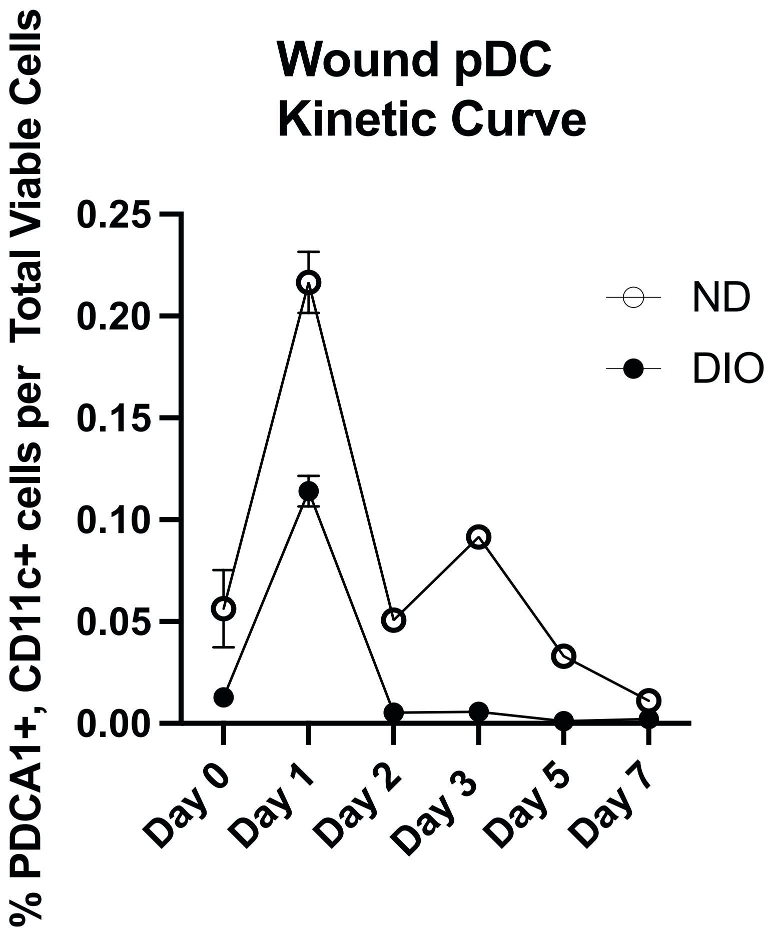

Supplemental Fig 7. Wound pDC quantity. Using flow cytometry, kinetic plot of non-diabetic (ND) and diabetic (DIO) wound pDCs from 6mm wounds days after wounding (N=5/group, pooled and repeated in triplicate).

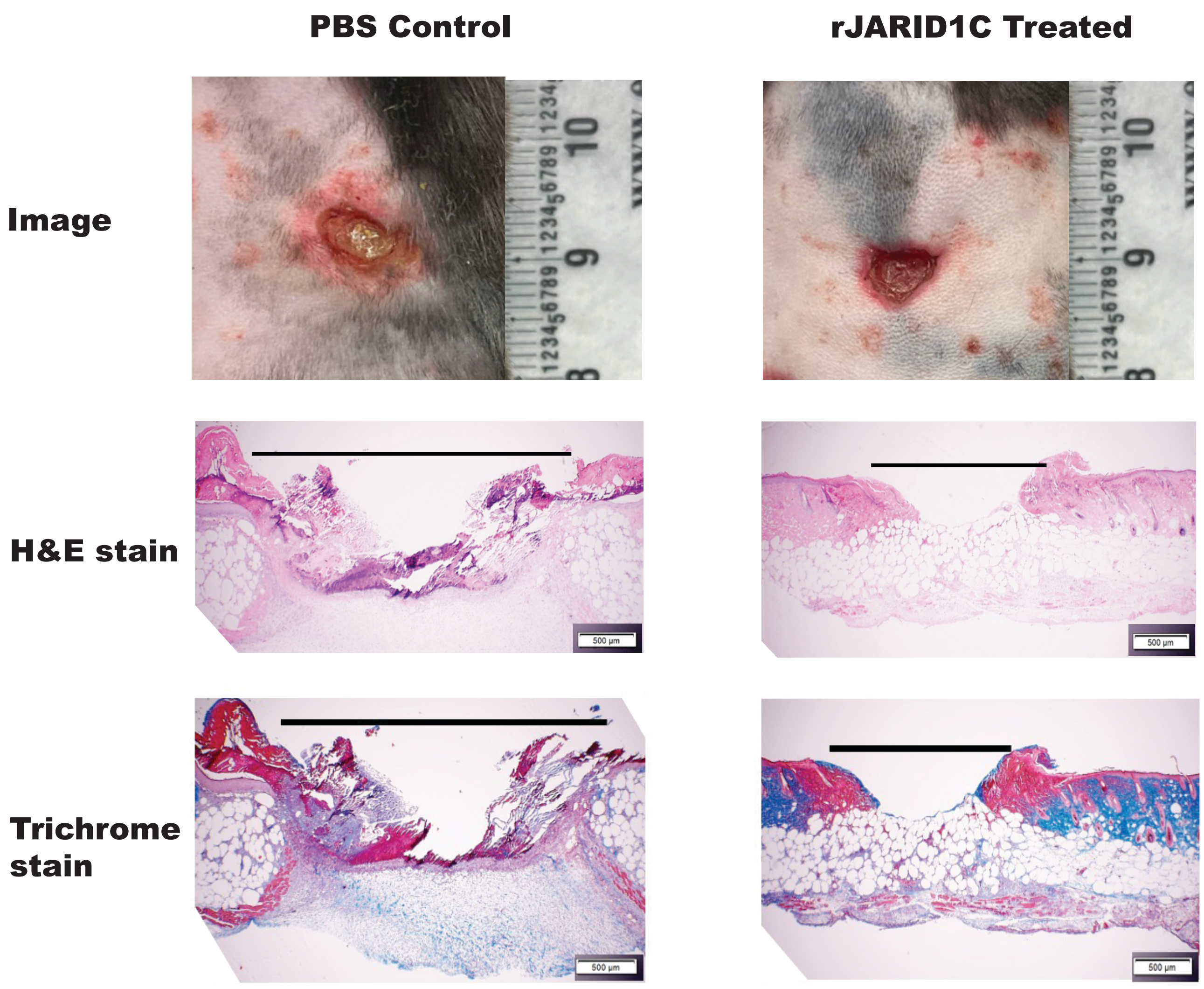

Supplemental Fig 8. rJARID1C supplementation on DIO wound healing. 6mm punch wounds were created on the dorsum of DIO mice and harvested on wound day 5 following peri-wound PBS injection (control) or recombinant JARID1C injection (1 µg/kg) on days 0 and 1. Images obtained on wound day 3. Representative histologic H&E stain and trichrome staining (collagen) is shown on day 5 wounds. Wound sizes are represented with line bars that span wound edges. Scale bars are shown. N = 4 wounds/mouse
